# Supplementary material for: Are carbon emissions trading and green financial instruments synergistic? -Comprehensive quantitative research based on content analysis
Source: PLoS One. 2024 Mar 7;19(3):e0298601. doi: 10.1371/journal.pone.0298601 (PMC10919601; doi:10.1371/journal.pone.0298601)
Supplement: S1 Data — (ZIP) [file pone.0298601.s001.zip › Data/地级市Z_F工作报告.zip!/╝¬┴╓/═¿╗»╩╨/└┤╫╘╣1⁄2╓┌║┼í╛┬φ┐╦╩2╛▌═°í┐_2014.docx]

一、2013年工作回顾

　　2013年，在省委、省政府和市委的坚强领导下，在市人大、市政协的监督支持下，市政府团结带领全市人民，抓项目、兴产业、惠民生、促发展，圆满完成了市七届人大一次会议确定的年度任务，实现了经济社会平稳较快发展。预计：

　　地区生产总值突破千亿大关，实现1003亿元，增长10%;

　　固定资产投资完成930亿元，增长22.2%;

　　全口径财政收入实现114.7亿元，增长8.1%;

　　地方级财政收入实现72.1亿元，增长12%;

　　社会消费品零售总额实现382.5亿元，增长13.5%;

　　城镇居民人均可支配收入达到24450元，增长13.5%;

　　农民人均纯收入达到10124元，增长13%;

　　节能减排约束性指标完成了省下达任务。

　　一年来，主要做了以下工作：

　　(一)推进项目建设，增强发展能力。实施3000万元以上项目1167项、亿元以上项目305项、10亿元以上项目30项，新上项目数量占75%。四长医药工业园、桦树医药工业园、通化生物科技产业园等重点项目快速推进，东宝人胰岛素注射剂三期、通鑫3000吨玄武岩纤维一期工程、帝翔60万件刹车盘、华润10万吨啤酒扩建等项目如期竣工。制定实施了鼓励投资若干规定，招商引资到位资金924亿元，实际利用外资3.4亿美元，引进亿元以上项目336项。环球医药东北区采购平台、宣捷医用电子仪器、橡胶轮胎和威高、攀华、康恩贝等一批大项目和知名企业落户通化。集约节约利用土地，规范清理存量，合理使用增量，新增建设用地指标296.7公顷，供应土地588.7公顷。通化市和二道江区列入国务院《全国资源型城市可持续发展规划(2013—2020年)》，医药高新区即将晋升为国家级高新区，国家农业科技园区建设获得批准，国家知识产权试点城市通过验收。列入国家和省科技专项70项，累计8个项目进入全省“双十”工程，高新技术企业发展到30户。24户企业被认定为全省首批创新型科技企业，占全省的20.7%。与北京海淀区、中关村和我省“四校两所”签订了科技战略合作协议，全省首家中医中药产业院士工作站和镁合金应用技术研究所、专利技术展示交易中心建成揭牌。集安市、通化县、东昌区荣获全国科技进步考核先进县(市、区)。

　　(二)稳定工业增长，调整产业结构。预计规模以上工业实现产值1626亿元、增加值406亿元，分别增长20.2%和12.5%，增速列全省首位;实现利润72.6亿元，创历史新高。技改投资完成356亿元，新产品实现产值190亿元。规模以上工业企业发展到535户、净增22户。新创2件中国驰名商标。实现外贸进出口总额5.8亿美元。产业跃升计划深入推进，八大重点工业产业产值增速超过20%。出台支持医药企业发展若干意见，37户企业通过新版GMP认证。医药产业产值规模达到700亿元，比上年净增140亿元。通钢淘汰落后产能置换项目加快实施，1号和2号焦炉技术改造及高强度机械制造用钢建成投产。绿色食品、保健品和生物医药、新材料、先进装备制造、新能源、节能环保等战略性新兴产业长足发展。举办了全国葡萄酒行业年会，鸭绿江河谷葡萄酒文化产业园开工建设。实施龙湾旅游度假区等45个旅游项目，完成10个魅力乡村、30个环城度假村建设任务，高句丽世界文化遗产列为全国旅游知名品牌创建示范区，五女峰入选中国最美森林旅游景区，云霞洞晋升为4A级景区，接待国内外游客增长29.2%，旅游收入增长34.6%。欧亚购物中心二期工程建成运营，红星美凯龙家居广场、蔬菜综合批发市场等商贸建设项目进展顺利。房地产业有序健康发展。

　　(三)完善政策支持体系，突出发展民营经济。制定出台了加快民营经济发展政策措施50条，启动全民创业等五大工程。建成创业发展服务中心和中小企业、个体工商户孵化基地、青年科技创业孵化基地。争取省农联社85亿元民营企业信贷额度，市本级设立1亿元专项资金。预计年末金融机构贷款余额比上年增加80亿元，中小微企业贷款增长20%。通化化工等7户企业分别在上海股交中心和香港OTC市场挂牌融资，通葡股份、通化金马完成定向增发，小额贷款公司发展到66家。民营经济实现主营业务收入2239.2亿元、实缴税金77亿元，分别增长20.3%、16.1%，“三上”企业达到1157户。

　　(四)狠抓基础设施和城市建设，推进城镇化。通梅高速公路全线开工，通靖高速开始征地拆迁，通集高速通过国家立项批复，辉白高速正在进行初步设计，官道岭互通立交桥建成通车，新建、养护国省干线及农村公路395公里。通丹铁路客运通车。通化机场完成试飞，正在做通航准备。改造市区棚户区及危旧房134万平方米，完成“暖房子”工程收尾及周边区域改造85万平方米。市区搬迁、拆除D级危房和工矿棚户区、塌陷棚户区63.7万平方米。滨江东路、西路堤路工程基本完成。升级改造了3座浑江主桥，江南新桥开工建设。通化大街工程统筹推进，街路巷路升级改造、江南新区路网、绿化美化亮化等工程按期完工。完成调峰热源改造一期工程，新增供热能力100万平方米，新建13座换热站和40公里配套管网，105万平方米新楼入网供热。完成城区31.6公里给水管网及老城区三期供水管网改造，新增煤气用户8500户。完成二道江50公里老旧燃气管网改造。“气化通化”管道工程开工建设。更新公交车40台，新开和延伸公交线路3条。城市管理、交通管理、物业管理得到加强，市容市貌进一步改观。编制完成特色城镇化发展规划，制定创建绿色生态新城区实施意见，江南新区加快建设，金厂镇、山城镇列入全省首批城镇化建设试点。完成污染物减排和重点流域污染防治工程146项，农业源减排代表吉林省接受环保部检查，哈泥河饮用水源保护工作扎实推进。市区建成2个PM2.5大气自动监测站，空气环境质量优良天数达到338天。新增5个国家级生态乡镇、10个省级生态乡镇、33个省级生态村。通化县被认定为国家有机食品基地试点县、全省首个省级生态县。

　　(五)加快农村和县域发展，提升农业现代化水平。粮食生产战胜低温春涝等灾害实现“四连丰”，总产量突破36亿斤。完成20万亩农作物高光效栽培技术示范项目和40个万亩高产示范方建设任务。建成标准化牧业小区220个，畜牧业产值增长20%。农村土地整治和新菜田建设进展顺利。新增棚膜蔬菜4160亩，中药材、蓝莓等特色产业基地发展到42万亩，大米、板栗、五味子3个国家级农业标准化示范区通过验收。实施超千万元产业化项目20项，省级以上农业产业化重点龙头企业发展到64家，农产品加工量达到220万吨。绿色有机无公害农产品增加到310种。国内最大的人参交易市场建成使用，康美人参产业园一期工程投产，9种人参产品列为长白山人参品牌，人参产业产值增长35.7%。实施新农村建设项目2142项，264个农村环境连片整治项目顺利完成。启动第二批“百村示范、全面提升”工程，争取省级示范村115个。农村电网结构和供电能力大幅提升。江河整治、水库除险加固等70项水利工程按期完成，浑江干流市区段堤防工程全线完工、达到50年一遇防洪标准。植树造林11.3万亩，绿化村屯398个，连续59年无重大森林火灾。农业保险覆盖耕地214万亩，农民专业合作社发展到2706个。梅河口市和柳河县位列全省县域考评前十位并分获综合发展二等奖、三等奖，通化县、辉南县获综合发展进步奖，我市获组织推动奖。梅河口市列入全省深化扩权强县改革试点。

　　(六)保障和改善民生，发展各项社会事业。民生工作计划中的116件实事全面完成。城镇新增就业5.8万人，农村劳动力转移就业42.3万人。养老、失业、医疗、工伤和生育保险新增参保6.5万人，城乡居民养老保险实现制度全覆盖。企业退休人员月人均养老金增加171元，城市低保月标准、农村低保年标准分别提高到360元、2240元，新型农村合作医疗政府补贴人均增至280元。医疗、教育等低保救助进一步加大。市直实行了公务员绩效考核奖励，调整提高了行政事业单位个人取暖费补贴方式和标准。市区保障性安居工程开工20087套、133.7万平方米，为低保和低收入住房困难家庭新建公(廉)租房1252套、7万平方米，完成2800户无籍房确权工作。改造农村危房2.6万户。建成农村饮水安全工程210处，解决了10.1万人饮水安全问题。义务教育均衡水平稳步提高，学前教育、职业教育进一步发展。新建改建幼儿园54所，实施农村薄弱校改造和义务教育学校标准化建设项目28项，市直校舍建设和维修改造顺利完成。公共卫生服务均等化工作成效显著，基本药物制度全面推开，各类传染病得到有效控制。市中心医院外科大楼和儿童医院建设主体工程完工，村卫生室标准化建设走在全省前列。文化事业和文化产业蓬勃发展。市科技文化活动中心完成主体建设，体育场改造全面推进。建成社区文化活动室36个、农村文化大院325个，乡镇综合文化站和农村文化大院实现省级标准全覆盖。宝泉涌酒坊等7处遗存列为全国重点文物保护单位。话剧《青春的猜想》获省转企改制院团新创剧目一等奖。举办多个文化产品博览会等会展，文化企业发展到2486户。完成广播电视编播系统数字化改造。全民健身活动广泛开展，农村体育健身工程覆盖率达到48.3%，竞技体育获省级金牌64枚。民族团结进步和人口计生、妇女儿童、老龄、残疾人事业全面发展。

　　(七)做好安全稳定工作，维护社会平安和谐。面对极其特殊的安全生产形势，持续组织开展安全隐患大排查大整改，排查整改各类隐患27412处，完成了地方煤矿兼并重组，事故起数和死亡人数连续3年实现“双下降”。安全生产工作代表吉林省接受国务院安委会督察，受到充分肯定。全力抗击历史罕见的局地洪涝灾害，实现了无人员伤亡的目标，倒塌房屋、水毁公路等恢复重建工作走在全省前列。排查调处矛盾纠纷1.2万件，解决了一批信访问题。市长公开电话受理群众来电5.8万件。理顺社区管理岗位职责，99个社区全部建立公共事务服务中心。深入推进平安通化建设，刑事案件发案率下降24%、破案率上升27%，群众安全感进一步增强。开展食品安全和药品质量专项整治，食品药品安全监管成效明显。支持国防和军队现代化建设，军政军民团结更加巩固。国家安全、外事侨务、人防、气象、地震监测、红十字会等工作都取得了新成绩。

　　(八)加强政风建设，提高行政效能。认真贯彻中央八项规定，积极解决“四风一顽症”。严格落实廉政建设责任制，完善惩防体系，反腐倡廉工作取得新成效。加强住房公积金规范管理。清查党政机关办公用房70.8万平方米。机构编制和公用经费保持“零增长”。坚持依法行政、从严治政，政务公开、行政监察、审计监督深入推进。自觉接受市人大及其常委会的法律监督、工作监督和市政协民主监督，办结人大代表建议101件、政协委员提案182件。“六五”普法扎实推进。政府机构改革任务基本完成。市本级清理政府规范性文件122件，取消、下放和暂停行政审批项目44项，行政审批时限压缩68%，法治政府建设取得新成果。广泛开展群众评议活动，加强公务员队伍教育管理，严明工作纪律，规范办事程序，提高服务能力和办事效率，经济发展软环境得到进一步改善。

　　年初以来，我市被评为中国十佳食品安全城市、最具幸福感城市、最美丽城市候选城市、全国创新能力百强地级市和安全生产月活动先进单位，“暖房子”工程获“中国人居环境范例奖”，国有土地上房屋征收经验在全省推广，全省校车安全管理、城市社区文化建设和农村环境连片整治等现场会在我市召开，获得全省绩效考核评比优秀档次。

　　各位代表!在宏观经济下行压力增加的情况下，取得这些成绩极为不易。这是市委科学决策、正确领导的结果，是市人大、市政协有效监督、大力支持的结果，是全市上下艰苦创业、团结奋斗的结果。在此，我代表市政府，向全市各族人民致以崇高的敬意和衷心的感谢!向人大代表、政协委员，向各民主党派、工商联、无党派人士和各人民团体，向参与通化发展建设的国省属单位、驻通部队、武警官兵，向所有关心支持通化的同志们、朋友们表示衷心的感谢!

　　在总结工作的同时，我们也清醒地认识到，前进道路上还有不少困难和挑战，工作中还有不少差距和问题：经济总量仍然不大，产业结构尚需优化，尽管一些指标增速在全省位次靠前，但持续保持高位增长的难度较大;中心城市辐射带动力不强，城镇化基础薄弱;社会建设与管理面临一系列新问题新挑战，影响社会稳定的因素依然存在;部分公职人员缺乏责任感和担当精神，执行力、落实力不强，有的甚至违法违纪。对这些问题，我们一定高度重视，采取有力措施，认真加以解决。

　　二、2014年目标任务

　　2014年，市政府要紧紧抓住国家全面深化改革的重大历史机遇，坚持以科学发展观为指导，认真贯彻落实党的十八大和十八届三中全会、省委十届三次全会、市委六届三次全会精神，以全面深化改革为统领，以保持经济平稳较快增长为重点，以调结构、转方式、增效益为主线，以保障和改善民生、增进人民福祉为根本，着力推动创新发展、统筹发展、绿色发展、开放发展、安全发展，大力推进工业化、信息化、城镇化和农业现代化，坚定不移抓项目、兴产业、惠民生、促发展，全面加强经济建设、政治建设、文化建设、社会建设和生态文明建设，为通化科学发展、加快振兴，全面建成小康社会奠定坚实基础。

　　主要预期目标：地区生产总值增长10%左右，财政收入、城乡居民收入与经济同步增长，固定资产投资达到千亿以上规模，社会消费品零售总额增长12%左右，城镇登记失业率控制在4.5%以内，节能减排完成省下达指标。实际工作中，要争取各项指标实现更大幅度增长。

　　(一)进一步加大投资工作力度。继续实施投资拉动，加快项目建设，做大经济总量。围绕重点产业、基础设施、改善民生、生态环保“四大板块”，新建续建3000万元以上项目1000项以上、亿元以上项目310项、10亿元以上项目30项。开工建设万通筋骨片扩能改造、莱福卡医疗电子仪器、大连船舶配套制造产业基地等新项目，加快实施四长医药工业园、桦树医药工业园、祥元镁制品产业园、帝翔汽车配件产业园、亚泰新型建材产业园等续建项目，推动通化生物科技产业园、通钢新2号高炉和3号转炉、玖鑫玄武岩纤维及制品等重点项目建成投产。强化项目管理和服务保障，完善项目推进责任制，做好土地、林地征用、环评和被征地农民补偿安置等工作，探索创新重大项目融资模式，提高项目质量。紧盯国家产业政策和投资导向，做好“十二五”后期和“十三五”项目准备，全年力争谋划亿元以上新项目150项，积极培育中小项目，为增强后劲打好基础。

　　(二)促进工业增长和产业转型升级。继续开展“工业企业服务年”活动，加强银企对接、产需衔接、要素保障和生产运行组织，扶持八大工业产业和百户重点企业、百种重点产品释放产能、扩产提效，推动现有企业同大企业、大集团嫁接改造、整合重组，进一步壮大实体经济，确保工业提速增效。规模以上工业总产值、增加值分别增长15%和11%，规模以上工业企业力争达到550户，产值超亿元企业力争达到260户。坚持信息化与工业化融合，推进信息技术在关键环节的集成应用，力争在设计研发智能化、生产过程自动化、经营管理信息化等方面取得突破。技改投资增长15%，新产品产值率超过12%。

　　实施支柱优势产业跃升、战略性新兴产业培育、特色资源产业提升计划，推动产业向集群化、高端化、规模化、品牌化转型升级。发挥医药高新区的政策集成优势，推进医药产业资源整合、药品研发创新和技术转让及园区化、产业化建设，吸引国内外知名企业和高端品种集聚，进一步提高医药产业的集聚度、丰厚度。完成26户医药企业新版GMP改造。支持服务通钢实施装备技术升级改造和转型发展，推动钢铁与地方关联产业协作配套，加快建设“五鸭铁”兴钢新区和钢材深加工项目，争取冶金产业企稳回升。实施山珍食品、绿色饮料等深加工项目，推进食品饮品生产基地建设，开发长白山特色食品资源，扶持壮大绿色食品产业。推动保健品产业加快发展。抓好鸭绿江河谷葡萄产业带建设，支持通葡、通天等骨干企业发展。开工建设葡萄酒主题文化公园，建成汇源葡萄酒庄及生产基地。加快发展生物医药、新材料、先进装备制造、新能源、节能环保等战略性新兴产业，培育医疗器械、医疗技术集成、医疗康复等健康产业集群。规划建设装备制造产业园。加强地质勘查，有序开发矿产资源。完成柳河、通化县石油项目勘探。加强医药、食品、机械制造等行业标准化建设和质量监管，实现生产抽验检验全覆盖。

　　推进服务业跨越发展。新建义乌城市综合体，建成红星美凯龙家居广场，加快欧亚、中东等商圈建设和业态升级，把江南新区打造成为区域性商务中心、消费中心。启动长白山医药物流中心二期工程，促进通丹陆港封关运营。建设通化大米、土特产品、成品粮油等区域批发市场。加快建设“数字通化”、“智慧通化”，积极发展电子商务、数据平台等现代服务业。规范发展房地产市场，保障群众住房需求。实施旅游产业壮大计划，推进白鸡峰国际养生度假区等50个景区景点建设，积极开通集安赴朝游和通丹铁路旅游专线，启动通丹旅游一体化合作机制，开发中远程客源市场，扩大通化旅游影响力。发展沟域旅游、乡村旅游和冬季旅游，继续推进魅力乡村、环城度假村建设。规划建设养老休闲度假基地。旅游收入增长35%。实施文化产业提速计划，开工文化旅游服务、科技文化创意等产业基地建设项目，加快培育“国药”、“国酒”、“国参”、“国砚”和剪纸刻纸等文化品牌，建成松花砚(石)门户网站和销售平台，扶持壮大一批特色鲜明的文化产业示范基地、示范园区和文化领军企业。文化产业增加值增长30%。做好全国第三次经济普查工作。

　　(三)全面深化改革、扩大开放。按照国家和省里的顶层设计、总体规划、改革路径和时间表，从市情实际出发，积极稳妥推进全面深化改革各项工作，构建充满活力、富有效率、更加有利于科学发展的体制机制。围绕转变方式、完善市场体系深化改革：实施负面清单管理，负面以外的内外资投资项目均可准入，改“严进宽管”审批制为“宽进严管”备案制;放宽工商登记条件;建立健全市场导向机制，加快科技成果产业化;构建新型农业经营体系，鼓励承包经营权向专业大户、家庭农场、农民合作社、农业企业流转，发展多种形式规模经营;鼓励社会资本参与市政基础设施建设运营，提升供水供气供热等服务水平;统筹推进财税、土地、金融等改革。围绕转变政府职能深化改革：进一步清理规范行政审批事项，优化政府职能配置、工作流程，切实简政放权;制定政府向社会力量购买服务具体实施办法，推动公办事业单位与主管部门理顺关系和去行政化;建立导向明确、指标科学的考核体系，加大生态效益、科技创新、安全生产和劳动就业、居民收入、社会保障等指标的权重。围绕健全城乡发展一体化体制机制深化改革：完善被征地农民社会保障制度;拓宽农村资源变资产、资产变资金途径;推进城乡最低生活保障制度统筹发展;推行进城落户农民养老保险，完善城镇化相关配套政策;落实财政转移支付同农业转移人口市民化挂钩机制，逐步把符合条件的农业转移人口转为城镇居民。围绕促进社会事业发展和创新社会治理深化改革：鼓励社会力量兴办教育;完善文化市场准入和退出机制;着眼解决影响和谐稳定的问题推进相关改革，创新有效预防和化解社会矛盾体制，促进新形势下社会组织健康发展。抓好特色示范镇建设、土地确权登记和林地、参地物权融资等试点。积极争取国家和省里的改革试点政策和资金、项目支持，推动改革实现突破。

　　全方位推进开放发展，努力把通化打造成为近海门户城市、沿边沿线开放城市。加快通丹经济带建设，密切对接长吉图和辽宁沿海经济带，深化与东北东部城市合作交流。加快集安边境经济合作区建设，争取鸭绿江界河大桥尽快联运通行。加快开放平台建设，推动各类开发区和特色园区转型升级、加快发展。医药高新区地区生产总值增长30%以上，开工、投产超亿元项目40项以上。加强出口基地建设，扩大对外贸易，实现外贸进出口总额6.2亿美元。突出产业招商、园区招商、集群招商，引进更多的战略投资者。加强与发达地区产业对接互动，吸引优质要素向通化集聚，在承接产业转移中实现加快发展。引进亿元以上项目360项，引进资金1000亿元，实际利用外资3.7亿美元。

　　(四)加快科技创新。强化自主创新，构建以企业为主体、市场为导向、产学研相结合的科技创新体系。启动百户创新企业集群工程，新培育高新技术企业3户、省科技型和创新型企业8户，创新型企业达到91户。抓好企业技术中心建设升级和认定工作，提高现有34个国家和省级企业技术中心自主研发能力。完善科技创新服务平台，加快院士工作站和科技孵化器建设，与中关村和高等院校、科研机构搞好合作对接，促进技术集成、成果转化。推动医药高新区、农业科技园区创新发展，启动建设中关村吉林科技产业园，规划建设吉林省高新技术特色产业基地。申报国家和省科技专项60项以上，争取2个项目进入全省“双十”工程。争创国家知识产权示范市。实施质量兴市和商标品牌战略，新创一批驰名商标、名牌产品和地理标志保护品种。完善科技人才激励政策，加大人才开发引进力度，支持科技人员开展科研、领办科技企业。通化科技专家公寓投入使用。

　　(五)强力推动特色城镇化。实施特色城镇化发展规划和十年生态园林城市规划，加快建设东北东部区域中心城市、中国北方生态宜居山水城市、国内重要的健康产业基地。坚持规划引领、产业支撑、政策集成、开放融资，突出渐进性、多样化、集约型、可持续，着力构建中心城市、县城、重点建制镇规模层级有序、功能优势互补、空间布局合理的城镇体系。抓好金厂镇1个城镇化建设省级试点和江南新区、湾湾川生态新城2个城镇化立体试验区，塑造和展示特色突出、品位提升、产业集聚、功能完善、生态和谐、环境优美的城镇化形象。规划建设通化至三源浦城港经济带，打造产业集聚走廊、城乡安居走廊。完善城镇基础设施、生活设施、娱乐休闲文化设施，强化城镇管理，促进城镇化与工业化、信息化、农业现代化相互融合，提高城镇化质量和水平。推动县城和城乡结合部城镇化，在各县(市)选定1个示范镇、2—3个3万人以上重点镇先行先试，带动全市城镇化加快推进。

　　加大中心城市建设力度，提升城市品位和承载辐射功能。市区城建计划投资55亿元。实施危房和新一轮各类棚户区改造，市区改造棚户区140万平方米，全面完成D级危房和塌陷棚户区改造。完成滨江东路江南段和滨江西路江北段续建工程，完善江南新区路网等基础设施。江南新桥建成通车。升级改造玉皇山公园、滨江公园，新建江南新桥景观公园。实施街路绿化美化彩化工程，推进滨江沿河、街路巷路亮化升级和楼道照明设施改造。抓好住宅小区综合整治，对不达标的小区进行环境设施改造。完成调峰锅炉扩容改造，新增供热能力100万平方米;新建换热站6座，铺设一次网15公里;改造陈旧供热管网40公里;启动新热源厂工程。实施“干净水”工程，对二次供水泵房进行整合和集中统一管理，保障居民用水和水质安全。延伸改造供水管线45公里。铺设改造燃气管线11公里，新增燃气用户6000户。实施公交便民工程，更换新型节能环保公交车50台，新开公交线路5条。加快停车场等公共交通基础设施建设，积极解决部分街路停车难和高峰拥堵问题。加强物业标准化管理。推进国家卫生城市、环保模范城市、园林城市和全国文明城市“四城”联创，开展乱堆乱放、占道经营、户外广告、犬类扰民造脏、破坏市政设施等专项整治，提高城市精细化管理水平。

　　(六)做大做强民营经济。推进“全民创业、招商引资、集群发展、市场培育、素质提升”五大工程，发展百姓创业经济，培育更多充满活力的市场主体，加快建设民营经济强市。落实各项扶持政策，加大财税支持力度，完善贷款担保体系，加强创业服务中心和孵化基地建设，创造中小微型企业发展良好环境。抓好企业上市，争取更多企业在上海股交中心和“新三板”挂牌融资。筹划发行城镇化集合债券，探索发行各类基金、企业债券、融资服务等金融业态，直接融资增长20%。支持城市商业银行、村镇银行和小额贷款公司发展。组建农村商业银行。新增贷款力争超过100亿元。民营经济主营业务收入增长20%，实缴税金增长17%，民营企业数量突破1万户，“三上”企业超过1200户。

　　(七)推进县域和农业农村发展。抓好东昌医药和镁合金产业基地、集安人参及农特产品精深加工、辉南医药包装和钢铁深加工、柳河医药及特色产品加工、通化县长白山药谷等园区建设，推动二道江实施产业创新、转型发展，县域工业投资、工业产值均增长25%以上。科学调整种植结构，继续实施农作物高光效栽培技术示范项目，5项重大粮食增产技术推广面积达到400万亩以上。统筹规划黄金水稻带，整合打造组织化、标准化、品牌化程度较高的通化精品大米。推进国家农业科技园区建设，搞好主核心区和“四园一中心”项目实施。开工建设卓越智能孵化养殖等20项产业化重点项目，吉煤特色农牧产业基地熟食加工项目建成投产。新建200个标准化牧业小区，畜牧业产值增长17%。实施特色产业基地建设提速工程，中药材、食用菌、蓝莓等基地面积发展到50万亩，园艺特产业产值增长12%以上。推进人参种植规范化、质量标准化、加工精深化、产业集约化，新建续建康美人参产业园、青山人参科研基地和精深加工等一批超亿元人参产业项目，完善人参电子交易等服务平台，开发人参新资源食品，促进人参产业在全省率先发展。人参产业产值增长25%。落实林业产业倍增计划，提高林业经济效益，确保连续60年无重大森林火灾。实施“菜篮子”工程，新建棚膜蔬菜4000亩，蔬菜种植突破35万亩。加强动植物疫病防控，推进农业标准化生产。新创绿色有机无公害农产品标识20个以上，申报国家有机食品基地3个、试点县1个。完善农村流通服务网络，组织农超对接，降低农产品流通成本。实施新农村建设项目2150项，开展“百村示范、全面提升”和“清洁文明提升”工程，争取115个示范村全部达到省级验收标准。

　　(八)抓好基础设施和生态文明建设。通梅高速公路完成路基、桥涵和隧道主体工程，开工建设通集、通靖、辉白高速公路。完成通快一级路拓宽改造，加快建设通化大街与通集高速公路立交连接工程。争取四平至通化至松江河快速铁路工程尽快启动。通化机场通航。改造10千伏供电线路7条、低压台区46座。加快建设望江楼和长川电站。抓好江河堤防整治、水库除险加固等73项水利工程。实施城市供水水源综合改造工程，争取扩容改造桃园水库，筹建从水库直接取水的第二净水厂，规划建设葫芦套和长流工业园，统筹解决市区供水保障、饮用水源保护、防洪安全和发展空间等问题。筹建市区应急备用水源。启动城市地下管网改造，力争用3年时间完成现有老旧管网更新改造。加快实施“气化通化”工程。

　　加强生态资源保护，建设天蓝地绿水净的美好家园。抓好哈泥河流域生态环境保护，完成辉发河、浑江等流域的18项污染防治项目。启动穿城水系和水域景观提升工程，推进周山沟河及公路、铁路沿线环境治理，打造周山和沿河沿线生态景观带。造林绿化10万亩，标准化绿化村屯48个。推进林地清收和退耕还林，依法打击毁林犯罪。搞好节能降耗和减排治污，加强大气污染综合治理。开工垃圾焚烧发电工程，筹建餐厨垃圾处理厂。实施115个农村环境连片整治示范项目，搞好农村垃圾和污水处理。加快推进二道江矿山地质环境综合整治。加强尾矿库、老矿山和地质灾害普查治理。建立健全能源、水、土地节约集约使用制度。抓好生态市建设，创建省级生态村30个、生态镇5个，争取通化县成为国家级生态县。

　　(九)协调发展各项社会事业。继续实施学前教育三年行动计划，加强幼儿园建设管理。加快农村薄弱校改造和义务教育学校标准化建设，推进义务教育均衡发展，规划完善教育布局，均衡配置教育资源。大力发展现代职业教育，深化产教融合、校企合作。支持通化师院参与地方发展建设。优化整合医疗资源，加快乡镇卫生院和村卫生室标准化建设，扩大公共卫生服务和基本药物制度覆盖面，加强重大传染病防控，增强医疗卫生服务保障能力。全面实施国家免费孕前健康检查。推进文化惠民工程，加强地域历史文化资源建设，培育名人名师名家名品，完善城乡公共文化服务体系。创建中国书法城。实施自安山城、万发等遗址建设保护工程，启动全国第一次可移动文物普查，做好国家级非物质文化遗产申报工作。广泛开展文化活动，繁荣文艺创作，打造艺术精品。新建改造一批城区健身带和农村文体广场。打好第十七届省运会。实施养老服务网络建设工程。维护妇女儿童权益。发展残疾人事业。推动民族团结进步和民族村镇发展。推进军民融合发展，扎实创建全国双拥模范城“八连冠”。

　　加快社会事业基础设施建设。市区重点抓好十个项目：1.科技文化活动中心投入使用;2.完成十三中、二十一中新建校舍搬迁，筹划一中校舍改造;3.推进中医院江南迁址工程，扩建结核病防治所业务用房;4.新建江南书法文化主题公园;5.启动建设全民健身中心;6.完成体育场改造工程;7.实施广播电视数字化改造，建设数字影院;8.建成公办社会福利服务中心;9.蔬菜综合批发市场迁址扩建工程建成开业，提升改造新站、集贸等区域的农贸市场;10.新建标志性公共候车亭20个。

　　(十)全力保障和改善民生。制定实施第五个民生工作计划，在富民增收、创业就业、社会保障等方面办100件左右实事。完善企业职工工资正常增长和支付保障机制，健全农民增收长效机制。城镇新增就业5.5万人，农村劳动力转移就业32万人。按政策提高基本养老金、失业保险金、城乡低保、城镇居民基本医疗保险、新型农村合作医疗补助标准和低保对象大病医疗救助标准。巩固提高城乡养老保险参保覆盖范围，确保各项养老金按时足额发放。完善社会救助体系，加大城乡低保和农村五保供养对象、残疾人等特困群体的帮扶救助力度。推进保障性安居工程，市区建成廉租房、公租房3.8万平方米，为1.6万户低收入家庭发放住房租赁补贴，完成1000户无籍房确权工作。改造农村危旧房5000户。建成农村饮水安全工程106处，解决4.3万农村人口饮水安全问题。

　　改进社会治理方式，维护社会稳定。推动系统治理、依法治理、综合治理、源头治理，继续实施重大决策社会稳定风险评估，健全基层综合服务管理平台。认真做好信访工作，建立畅通有序的诉求表达、心理干预、矛盾调处、权益保障机制，使群众问题能反映、矛盾能化解、权益有保障。大力开展平安通化创建活动，完善立体化社会治安防控体系，依法严密防范和惩治各类违法犯罪。强化边境管理，维护边境稳定。深入开展安全隐患大排查大整改，全面推进安全生产信息化、标准化和安全监管网格化、社会化“四化”融合，属地监管、行业监管、综合监管“三位一体”的监管机制建设，着力健全安全生产长效机制，严防重特大安全事故发生。加强校车和校园安全管理。加强物价监测监管。提升食品药品安全问题的事前防范能力，建立严格的覆盖全过程的监管制度，保障群众饮食用药安全。

　　三、加强政府自身建设

　　以开展党的群众路线教育实践活动为契机，创新政府服务和管理，建设效能政府、法治政府、服务政府、清廉政府。

　　改进工作作风。牢固树立正确的政绩观、事业观，立足当前，着眼长远，打牢基础。加强学习和调查研究，继续精减会议和文件，集中精力抓落实。坚持深入基层，夙夜在公，勤勉尽责，敢于较真，敢于碰硬，知难而进，迎难而上。集聚机遇能量，放大优势因素，激发创造活力，争创一流业绩。强化绩效管理，健全问责制度，缩短流程，精减环节，提高效能。

　　密切联系群众。尊重群众，贴近群众，千方百计听民声、纳民意、解民忧、惠民生。完善直接联系和服务群众制度，思利民之策，办利民之事，兴利民之举。拓宽社情民意反映渠道，加强电子政务、网上办公、政务大厅、政府网站、市长公开电话、信访接待等服务平台建设，促进政务服务更加便民、高效。

　　坚持依法行政。自觉接受市人大及其常委会的法律监督、工作监督，定期向人大报告工作，认真落实人大及其常委会的决议和决定。积极支持市政协履行政治协商、民主监督和参政议政职能，推进协商民主广泛多层制度化发展，增加协商密度，提高协商成效。办理好人大代表议案、建议和政协提案。推行政府及其工作部门权力清单制度，依法公开权力运行流程，规范工作程序。加强重点领域基层执法力量，严格规范公正文明执法。深入开展“六五”普法教育。进一步解决不作为、乱作为等问题，加大涉软案件查处力度，优化经济发展软环境。

　　加强廉政建设。广泛开展廉洁从政教育，筑牢拒腐防变的思想防线。全面落实廉政建设责任制，用制度管事、管权、管钱、管人。加强行政监察、审计监督，健全财务预算核准制度，规范政府性资金管理和使用，深入推进“五权”工作，纠正部门和行业不正之风，严肃查处违纪违法案件。艰苦奋斗，厉行节约，严控“三公”经费支出、楼堂馆所建设和机构编制。

　　各位代表!全面深化改革、加快振兴发展的号角已经吹响，我们任重道远。让我们在市委的坚强领导下，在市人大、市政协的大力支持下，锐意进取，攻坚克难，扎实做好新一年工作，为全面建成小康社会，让全市城乡居民生活得更加美好而努力奋斗!
